# Border Lines of Knowledge, in Some Provinces of Medical Science

**Published:** 1862-03

**Authors:** 


### Book Notices.

Border Lines of Knowledge, in some Provinces of Medical Science. An Introdutory Lecture, delivered before the Medical Class of Harvard University, November 6th, 1861. By Oliver Wendell Holmes, M. D., Parkman Professor of Anatomy and Physiology. Boston: Ticknor and Fields. 1862.

In one of the characteristic lectures of Horace Mann, he introduces, very happily, the following figure:—"The Germans and French have a beautiful phrase, which would enrich any language that should adopt it. They say, '*To orient;*' or '*to orient one's self!*"

"When a traveller arrives at a strange city, or is overtaken by night, or by storm, he takes out his compass and learns which way is the East, or Orient. Forthwith all the cardinal points—east, west, north, south—take their true places in his mind, and he is in no danger of seeking for the sunset or the pole-star in the wrong quarter of the heavens. *He orients himself.*"

In the little volume of eighty pages before us, Prof. Holmes has furnished, not only the student, but the practitioner, a compass, whereby he that cares to accurately define his position with reference to what has been accomplished, may orient himself, as he starts in quest of the vast spaces, yet enclosing infinite unknown details in Chemistry, Anatomy and Physiology. These, *par excellence:* for when HOLMES assumes to treat of the healing arts — medicine and surgery — when he leaves his closet for the sick-chamber, and drops his scalpel and lens, with which he has been questioning the inanimate fibre of the cadaver, to note the living wheels of the pulsing, breathing organism, rudely jarring and clashing in the disorder of disease, or broken in the shock of wounds and lesions, he, himself, needs *to orient* — at least, he forgets to allow for the variation of his magnetic needle — a variation in his scientific system, very evidently increasing in a swifter ratio, than the 4′ annually allowed the symbol of constancy and guidance.

Dr. HOLMES is, as every man must be, who devotes himself exclusively to the closet-study of theoretical or preliminary branches, wanting in practicability; wanting in that confidence and knowledge which comes only by experience, by the application in daily practice, of the theories and speculations of the laboratory and dissecting room. This is the *experimentum crucis*, by which remedies—surgical and medical—must stand or fall; and not all the polished satire, brilliant antitheses, and poetic fancy, of even a HOLMES, can do more than evanescently swerve the decision.

The world of Medical Science is boundless; and if, in some departments, Prof. HOLMES' sway is undisputed, there is no danger as yet, of his being reduced to the lachrymose condition of Alexander, or of reversing the saying of the Father of medicine, which he avers he cannot forget—*Ars longa, judicium difficile.* Let the glory he has reaped with the scalpel and the poet's plume satisfy him, then, without essaying the wholesale destruction of the labors of such men as Pereira and Dunglison, Wood and Bache, Headland, Christison, Trousseau, and scores of others, not less eminent in their respective spheres, than the Anatomist of Harvard in his.

Of course, every page of the *Border Lines*, sparkles with gems of poesy and fancy, until the hard lines of science assume the brilliant hues of fairy-tales, and truth, stranger than fiction, comes near to losing her own identity. We quote, here and there, from the well-printed page:—

It (Chemistry) is accomplishing wonders before us every day, such as Arabian story-tellers used to string together in their fables. It spreads the sensitive film on the artificial retina which looks upon us through the optician's lens for a few seconds, and fixes an image that will outlive its original. It questions the light of the sun, and detects the vaporized metals floating around the great luminary,—iron, sodium, lithium, and the rest,—as if the chemist of our remote planet could fill his bell-glasses from the fiery atmosphere.* It lends the power which flashes our messages in thrills that leave the lazy chariot of day behind them. It seals up a few dark grains in iron vases, and lo! at the touch of a single spark, rises in smoke and flame a mighty Afrit with a voice like thunder and an arm that shatters like an earthquake. The dreams of Oriental fancy have become the sober facts of our every-day life, and the chemist is the magician to whom we owe them. * * * * * *

These facts of allotropism have some corollaries connected with them rather startling to us of the nineteenth century. There may be other transmutations possible besides those of phosphorus and sulphur. When Dr. Prout, in 1840, talked about azote and carbon being "formed" in the living system, it was looked upon as one of those freaks of fancy to which philosophers, like other men, are subject. But when Professor Faraday, in 1851, says at a meeting of the British Association, that "his hopes are in the direction of proving that bodies called simple were really compounds, and may be formed artificially as soon as we are masters of the laws influencing their combinations,"—when he comes forward and says that he has tried experiments at transmutation, and means, if his life is spared, to try them again,—how can we be surprised at the popular story of 1861, that Louis Napoleon has established a gold-factory and is glutting the mints of Europe with bullion of his own making? * * * * * *

This is the place, if anywhere, to mention any observations I could pretend to have made in the course of my teaching the structure of the human body. I can make no better show than most of my predecessors in the well-reaped field. The nucleated cells found connected with the cancellated structure of the bones, which I first pointed out and had figured in 1847, and have shown yearly from that time to the present, and the *fossa masseterica*, a shallow concavity on the ramus of the lower jaw, for the lodgment of the masseter muscle, which acquires significance when examined by the side of the

* Scientific Annual for 1861.—FAIRBAIRN'S Address before the British Association, 1861.

deep cavity on the corresponding part in some carnivora to which it answers, may perhaps be claimed as deserving attention. I have also pleased myself by making a special group of the six radiating muscles* which diverge from the spine of the axis, or second cervical vertebra, and by giving to it the name *stella musculosa nuchæ.* But this scanty catalogue is only an evidence that one may teach long and see little that has not been noted by those who have gone before him. * *

It is curious that the Japanese should have anticipated Europe in a kind of rude regional anatomy. I have seen a manikin of Japanese make traced all over with lines, and points marking their intersection. By this their doctors are guided in the performance of acupuncture, marking the safe places to thrust in needles, as we buoy out our ship-channels, and doubtless indicating to learned eyes the spots where incautious meddling had led to those little accidents of shipwreck to which patients are unfortunately liable. * * *

But the great triumph of the microscope as applied to anatomy has been in the resolution of the organs and the tissues into their simple constituent anatomical elements. It has taken up general anatomy where Bichat left it. He had succeeded in reducing the structural language of nature to syllables, if you will permit me to use so bold an image. The microscopic observers who have come after him have analyzed these into *letters*, as we may call them,—the simple elements by the combination of which Nature spells out successively tissues, which are her syllables, organs which are her words, systems which are her chapters, and so goes on from the simple to the complex, until she binds up in one living whole that wondrous volume of power and wisdom which we call the human body.

The alphabet of the organization is so short and simple, that I will risk fatiguing your attention by repeating it,—according to the plan I have long adopted.

A. Cells, either floating, as in the blood, or fixed, like those in the cancellated structure of bone, already referred to. Very commonly they have undergone a change of figure, most frequently a flattening which reduces them to scales, as in the epidermis and the epithelium.

B. Simple, translucent, homogeneous solid, such as is found at the back of the cornea, or forming the intercellular substance of cartilage.

---

* *Rectus capitis posticus major, obliquus capitis inferior* and *semispinalis colli*, on each side.

C. The white fibrous element, consisting of very delicate, tenacious threads. This is the long-staple textile substance of the body. It is to the organism what cotton is pretended to be to our Southern States. It pervades the whole animal fabric as areolar tissue, which is the universal packing and wrapping material. It forms the ligaments which bind the whole framework together. It furnishes the sinews, which are the channels of power. It enfolds every muscle. It wraps the brain in its hard, insensible folds, and the heart itself beats in a purse that is made of it.

D. The yellow elastic, fibrous element, the caoutchouc of the animal mechanism, which pulls things back into place, as the india-rubber band shuts the door we have opened.

E. The striped muscular fibre, — the red flesh, which shortens itself in obedience to the will, and thus produces all voluntary active motion.

F. The unstriped muscular fibre, — more properly the fusiform-cell fibre, which carries on the involuntary internal movements.

G. The nerve-cylinder, a glassy tube, with a pith of some firmness, which conveys sensation to the brain and the principle which induces motion from it.

H. The nerve-corpuscle, the centre of nervous power.

I. The mucous tissue, as Virchow calls it, common in embryonic structures, seen in the vitreous humor of the adult.

To these add X, granules, of indeterminate shape and size, Y, for inorganic matters, such as the salts of bone and teeth, and Z, to stand as a symbol of the fluids, and you have the letters of what I have ventured to call the alphabet of the body.

But just as in language certain diphthongs and syllables are frequently recurring, so we have in the body certain secondary and tertiary combinations, which meet more frequently than the solitary elements of which they are composed.

* * * We do not know where we now stand in the hierarchy of created intelligence. We were *made* a little lower than the angels. I speak it not irreverently; as the lower animals surpass man in some of their attributes, so it may be that not every angel's eye can see as broadly and as deeply into the material works of God as man himself, looking at the firmament through an equatorial of fifteen inches' aperture, and searching into the tissues with a twelfth of an inch objective.

But if we continue in this manner, we shall get two-thirds of the volume in our pages; and then, if we did as we want to, would go back and add the remainder.

